# A novel algicidal properties of fermentation products from *Pseudomonas* sp. Ps3 strain on the toxic red tide dinoflagellate species

**DOI:** 10.3389/fmicb.2023.1146325

**Published:** 2023-04-17

**Authors:** Luwei Zheng, Hong Lin, Barathan Balaji-Prasath, Yuping Su, Ying Wang, Yi Zheng, Guanglang Yu

**Affiliations:** ^1^College of Environmental and Resource Science, College of Carbon Neutral Modern Industry, Fujian Normal University, Fuzhou, China; ^2^Fujian Key Laboratory of Pollution Control and Resource Recycling, Fujian Normal University, Fuzhou, China; ^3^Fujian Province Research Centre for River and Lake Health Assessment, Fujian Normal University, Fuzhou, China; ^4^Fujian Key Laboratory of Special Marine Bio-resources Sustainable Utilization, Fujian Normal University, Fuzhou, China

**Keywords:** *Pseudomonas*, dinoflagellate, algicidal bacteria, active substances, red tide

## Abstract

The viability of both China’s offshore fishing operations and the global marine fishing industry is threatened by the occurrence of red tides caused by *Gymnodinium catenatum* and *Karenia mikimotoi*. Effective control of these dinoflagellate-mediated red tides has become a pressing issue that requires immediate attention. In this study, High-efficiency marine alginolytic bacteria were isolated and underwent molecular biological identification to confirm their algicidal properties. Based on a combination of morphological, physiological, biochemical, and sequencing results, Strain Ps3 was identified as belonging to the species *Pseudomonas* sp. We examine the effects of algicidal bacteria on the red tide species *G. catenatum* and *K. mikimotoi* within an indoor experimental setting. Then gas chromatography– mass spectrometry (GC–MS) was used to analyze the structure of the algolytic active substances. This investigation demonstrated that with exposure to the algae-lysis experiment, the Ps3 strain has the best algae-lysis effect, with *G. catenatum* and *K. mikimotoi* reaching 83.0 and 78.3%. Our results from the sterile fermentation broth experiment showed that the inhibitory effect on the two red tide algae was positively correlated with the concentration of the treatment. At a treatment concentration of 2.0% (v/v), the 48 h lysis rates of *G. catenatum* and *K. mikimotoi* due to exposure to the Ps3 bacterial fermentation broth were 95.2 and 86.7%, respectively. The results of this study suggest that the algaecide may be a rapid and effective method to control dinoflagellate blooms, as evidenced by the observed changes in cellular morphology in all cases. In the ethyl acetate phase of Ps3 fermentation broth, the cyclic (leucine-leucine) dipeptide was the most abundant. The findings of this study contribute to our understanding of red tide prevention and control and provide a theoretical foundation for further research in this field.

## 1. Introduction

An expanding body of research has been dedicated to identifying strategies for preventing, managing, and mitigating harmful algal blooms (HABs), which are increasingly spreading and intensifying across different geographical locations (Anderson and Menden-Deuer, 2017; Shi et al., 2018; Baohong et al., 2021; Balaji-Prasath et al., 2022b). A red tide, which is alternatively referred to as a bloom, is a natural ecological phenomenon that occurs when high-density algae cells in seawater cause discoloration. In coastal farming regions, the eruption of red tides leads to the secretion of sticky substances by plankton blooms that attach to the gill tissues of fish, resulting in the death of numerous farmed fish and significant losses to fisheries (Pokrzywinski et al., 2017; Li et al., 2018). In the last decade, scientists have dedicated considerable efforts towards developing various techniques aimed at mitigating the frequency and negative impacts of red tides. These methods entail controlling the growth of harmful species and reducing the concentration of toxic substances (Visciano et al., 2016; Petropoulos et al., 2019; Ko et al., 2022). The factors that contribute to the development of red tide are multifaceted and varied. However, bacteria play a crucial role in the dissipation phase of red tide (Sun et al., 2016; Zohdi and Abbaspour, 2019). It is widely accepted that the bacterial community undergoes quantitative and qualitative changes during an algal bloom and may potentially exert a positive or negative influence on the regulation of algal growth. It is important to highlight that certain organisms, notably algicidal bacteria, have the ability to break down algae by either directly attacking their cells or indirectly through the secretion of compounds such as proteins, polypeptides, biosurfactants, amino acids, and antibiotics with algicidal properties (Zhuang et al., 2018).

The mitigation strategies for HAB species, including microbes such as bacteria, fungi, viruses, and grazers such as copepods, protozoa, and macrophytes have already been investigated and tested by many studies, which are crucial to understand the dynamics and succession of HAB species (Zheng et al., 2013). Several bacterial strains that can inhibit or kill HAB species have been isolated in studies focusing on algal-bacterial interactions. Currently, there is a lack of scientific understanding regarding the mechanism by which these compounds effectively eliminate their target algal species, specifically those associated with red tide, and the ecological function of algicidal bacteria (Meyer et al., 2017). Furthermore, the majority of algicidal bacteria exhibit efficient, algae-specific, and environmentally sound characteristics. These microorganisms employ two mechanisms to target algal cells, which include direct cell-to-cell contact (Li et al., 2016; Van Tussenbroek et al., 2017). Alternatively, these microorganisms may also employ the use of algicidal compounds, such as antibiotics, enzymes from actinomycete, and fungi, to indirectly mediate the interaction between microalgae and other microorganisms (Hussain et al., 2013; Feng et al., 2014; Balaji Prasath et al., 2021). Algicidal bacteria have complex and diverse active substances and show good algicidal activity in controlling harmful red tides. For example, the active substance of *Pseudomonas* to control *Karenia brevis* is saponin, and bacillamide in *Bacillus* showed 50.0% lysis rates in 6 h against *Cochlodinium polykrikoides* (McCoy and Martin, 1977; Jeong et al., 2003). Consequently, algicidal microorganisms and their associated compounds may serve as more effective and environmentally sustainable agents for managing HABs in the aftermath of marine calamities. The identification of multiple dominant species would serve as a valuable reference point for enhancing our comprehension of the incidence, prevention, and management of red tide. As a result, numerous red tide control experts have exerted considerable efforts in identifying efficacious alga-solubilizing bacteria and their potential bioactive compounds for the dissolution of algae. This study delves into the algolytic properties of *Pseudomonas* Ps3, an effective strain with algal lytic and inhibitory capabilities, to enhance its algolytic efficiency. The research provides a valuable technical reference for red tide management and highlights the potential of *Pseudomonas* Ps3 as a promising bacterial agent for controlling algal blooms.

## 2. Materials and methods

### 2.1. Species identification of *Pseudomonas* sp. Ps3

Conducting an examination of bacterial traits and features. This process entails the scrutiny of the physical attributes of bacterial colonies, in addition to utilizing both Gram staining and scanning electron microscopy methodologies to investigate their morphology and structure.

The strain was cultivated in a liquid beef paste medium until it reached the logarithmic phase. Under aseptic conditions, transfer 2.0 ml of the sample to a sterile centrifuge tube, seal it with a sealing film, refrigerate and send it to Beijing Ovison Genetics Co., Ltd. for 16S rDNA identification. The main steps include: (1) PCR amplification, followed by agarose gel electrophoresis of the amplified fragments; (2) purification of the PCR products; (3) BigDye®Terminator v3.1 sequencing reaction and purification; (4) sequencing data collection using the 3,730 xl.

The 16S rDNA sequence obtained from sequencing will be compared to the NCBI GenBank database using BLAST to select bacterial species with a similarity of 99.5% or higher, and to construct a phylogenetic tree to preliminarily confirm the bacterial genus.

### 2.2. Experimental procedure and calculation of algae dissolution rate

The harmful dinoflagellates, *G. catenatum* and *K. mikimotoi*, used in this study were obtained from the State Key Laboratory of Offshore Marine Environmental Science (Xiamen University). The cultures were maintained in a sterile L1 seawater medium at a temperature of 20°C, with a photon flux of 100 μmol photons·m^−2^·s^−1^ and a 14 h:10 h light: dark cycle, as previously described (Shi et al., 2013). The algicidal bacterium, Ps3, was cultured in beef extract peptone medium and amplified on a constant temperature shaker at 30 ± 1°C for 24 h. The bacteria were preserved with 50.0% glycerol at a ratio of 1:1. To prepare the algae sample for observation and counting, 500 μl of the shaken red tide algae culture solution were transferred into a 1.5 ml centrifuge tube, and Lugol’s reagent was added gradually to stabilize the algae. Subsequently, 100 μl of the resulting algae solution was carefully dispensed into an algae counting frame and examined under the Jiangnan BM2000 photo microscope. The counting process was repeated three times, and the observed relative error was within 5.0%. The bacterial solution was mixed with *G. catenatum* and *K. mikimotoi* at a volume ratio of 1.0% (v/v), and a control group was prepared using an equivalent amount of beef extract peptone medium. Samples were collected at predetermined intervals (4, 8, 12, 18, 24, 36, and 48 h), and three replicates were examined for each group to quantify the number of algal cells and the rate of algal dissolution. To ensure the validity of the results, all experimental procedures were conducted in a sterile environment to prevent bacterial contamination.

### 2.3. Determination of growth curve of algolytic bacteria

The plate colony counting method was used to count, and 1.0% of recovered Ps3 bacteria by volume were inoculated into a 100.0 ml medium and cultured in a constant temperature shaker at 30°C and 130 rpm. Samples were taken every 2 h from the same batch of the medium as control and measure the growth absorbance of the bacterial solution at OD600. At the same time, another 100 μl was taken every 4 h and diluted 10^−6^, 10^−7^, 10^−8^, and 10^−9^ layers were used for plate coating, three parallel samples. Then placed in a constant temperature incubator at 30 ± 1°C for 24 h after sealing. The bacterial population was quantified for each growth phase, and a growth curve for Ps3 was generated.

### 2.4. Experiment on the action mode of algolytic bacteria

After reaching the logarithmic phase, the Ps3 broth (SBS) was diluted to 1.0% by volume, inoculated into a fresh medium, and subsequently incubated in a constant temperature shaker for 24 h at 30°C and 130 rpm. The OD600 value of the bacterial broth was measured in real-time. After centrifugation at 10,000 rpm for 15 min, the supernatant and precipitated cells were collected separately. The supernatant was filtered three times with a disposable sterile filter tip of 0.22 μm pore size PES membrane to obtain the sterile fermentation broth of *Pseudomonas* Ps3 (S). The precipitated cells were subjected to vortex shaking with an appropriate amount of medium for 1 min, followed by centrifugation at 10,000 rpm to remove the supernatant. This washing process was repeated thrice, and the volume was adjusted to 5.0 ml with sterile water to obtain the bacterial suspension (BS). A fresh medium was used as the control. The prepared SBS, S, and BS were added at a 2.0% volume ratio to 2.0 ml of *G. catenatum* and *K. mikimotoi* during the growth period. Three parallel samples were taken and counted to determine the rate of algal lysis.

### 2.5. Experiment on influencing factors of algal dissolution in a bacterial fermentation broth

#### 2.5.1. Experiment on the effect of algae dissolution by the amount of bacterial solution

Ps3 was incubated in beef paste liquid medium at 30°C and 130 rpm for 24 h to prepare the Ps3 fermentation broth. The real-time OD600 value of the bacterial broth was determined. A culture medium was used as the control, and the Ps3 bacterial fermentation broth was inoculated into the algal broth of *G. catenatum* and *K. mikimotoi* at volume ratios of 0.1, 0.5, 1.0, 2.0, and 4.0%.At predetermined intervals, samples were collected and the rate of algae lysis was determined.

#### 2.5.2. Experiments on the effect of temperature on algae lysis

Given that the red tide *of G. catenatum* and *K. mikimotoi* in Fujian typically occurs between April and June, with actual water temperatures ranging between 15 and 25°C, we set up three gradient temperatures of 15, 20, and 25°C. Sterile Ps3 fermentation solution was injected separately at a volume ratio of 2.0%, with a light intensity of 3,000 lx and a light–dark ratio of 12 h:12 h. A medium blank control was used to generate three parallel samples, which were then counted under a microscope at regular intervals to calculate the algal lysis rate.

### 2.6. Component separation of fermentation broth

The control consisted of a medium that was prepared in the same batch, and 100.0 ml of cultured bacteria Ps3 was inoculated for 48 h. The OD600 value was measured with three parallel samples, and the Ps3 fermentation broth was obtained using a 0.22 μm acetate fiber filter membrane three times. The solvent was then dried by vacuum distillation at 80°C. Next, 1.0 ml of ethyl acetate was added, and the solution was oscillated in an oscillator at 130 rpm for 10 min. The solution was repeatedly dissolved in the same way 3 times, and finally, the volume was fixed to 10.0 ml, resulting in the ethyl acetate phase solution (A). The remaining phase was dissolved in 1.0 ml water in the oscillator at 130 rpm and oscillated for 10 min, repeated three times, and kept at a constant volume of up to 10.0 ml to obtain the remaining phase (B). Both components A and B were stored in a refrigerator at −4°C for later use.

### 2.7. Experiment of algae solubilization effect of different components

To establish a control, algae of *G. catenatum* and *K. mikimotoi* were subjected to a culture medium. Additionally, ethyl acetate, ethyl acetate phase solution (A), and residual phase (B) were added to the algae at concentrations of 0.1, 0.5, 1.0, 2.0, 4.0, and 5.0% (v/v). The incubation temperature was maintained at 20 ± 1°C, while the light intensity was set at 3000 lx, and the light–dark ratio was set at 12 h:12 h. At predetermined intervals, parallel samples were collected and the number of algal cells was quantified three times at 24 h to assess the impact of algae lysis and calculate the corresponding algae lysis rate.

### 2.8. Qualitative identification of the components of the fermentation broth of the bacteria

A sample of approximately 0.5–1.0 ml of ethyl acetate extract was taken in a sampling bottle, with pure ethyl acetate being used as a control. The composition and structure of the algae-solubilizing substances, as well as the percentage content of the main substances, were analyzed using an Agilent 5977/7890B gas chromatograph-mass spectrometer from Agilent.

### 2.9. Experiment to test the toxicity of aquatic organisms

*Pseudomonas* Ps3 organism acute toxicity experiments organism select common *Brachionus plicatilis*, *Artemia salina*, and *Oryzias latipes*. These three experimental organisms belong to different phyla and trophic levels in marine ecosystems and can comprehensively represent different groups of animals in the ocean. Table 1 outlines the sources and culture requirements of these experimental species. Prior to experimentation, all organisms are subjected to a 24-h period of starvation in seawater. Use a blank group that has not been inoculated with bacterial fermentation solution as a control, with 10 organisms randomly assigned to each experimental group. Parallel samples are established for each group to ensure accuracy and consistency. The Ps3 bacterial solution (1 × 10^7^ CFU/ml), in the stationary phase and at a volume ratio of 2.0%, is introduced into three experimental groups. Individual mortality is assessed every 24 h, with the criterion for death being the lack of response to external stimuli. The experiment is conducted under controlled conditions of a light intensity of 2,650 ± 100 lx, a light–dark cycle ratio of 12 h:12 h, and a temperature of 20 ± 1°C.

**Table 1:** The source and cultivation conditions of the test organisms.

Species	Source	Temperature	Conditions
*Brachionus plicatilis*	The Institute of Oceanology, Chinese Academy of Sciences	20 ± 1°C	Mature individual without an ovipositor
*Artemia salina*			Newly hatched fish (1–2 days old, body length < 1 mm)
*Oryzias latipes*			Healthy fish with sensitive response, normal appearance, and uniform body weight of 20 ± 2 mg at approximately 60 days old

### 2.10. Data processing

The growth rate of the microalgal culture is calculated according to (Balaji Prasath et al. (2021)) by the following equation


(1) $$R=\frac{\left(lnC_{2}-lnC_{1}\right)}{t_{2}-t_{1}}$$

C_1_ and C_2:_ represent the initial and current algal cell concentration, cell/ml; t_1_, t_2_: initial and current culture time, h; R: specific growth rate, d^−1^.

The formula for calculating the number of bacteria is:


(2) C=X×N×10

Where C represents the bacterial concentration of the sample (CFU/ml), X represents the dilution ratio of the sample, and N represents the number of bacterial colonies on the plate.

To determine the calculation of algae dissolution rate in all experimental groups, the following formula was used (Balaji-Prasath et al., 2022a).


(3) $$R=\left(\frac{C_{0}-C_{1}}{C_{0}}\right)\times 100\%$$

where C_1_-experimental is the algae density of the treated culture and C_0_-control is the algae density of the control culture, cell/ml.

## 3. Results

### 3.1. Molecular biology identification results

Ps3 bacterial colony exhibits a round morphology with a slight elevation and a diameter between 1 and 3 mm. The colony surface is smooth, moist, milky-white, and opaque (Supplementary Figure 1). The Gram staining of Ps3, illustrated in Supplementary Figure 2, revealed a blue color, indicating that it is a Gram-negative bacterium. It typically occurs in single rod-shaped or chain-like structures. Based on the Gram staining results and the physiological and biochemical properties of the bacteria, Ps3 was provisionally identified as a straight or slightly curved rod. Subsequently, scanning electron microscopy was employed to further observe the bacterial morphology, as depicted in Supplementary Figure 3. The individual Ps3 bacteria had sizes ranging from 1.5–1.7 μm in length and 1.1–1.3 μm in width, with multiple organisms adhering to each other without flagella. The presence of spores was not detected.

The 16S rDNA gene sequence of the Ps3 strain was amplified, resulting in a base pair count of 1,437 bp according to the identification results. The strain’s basic information and gene sequence was uploaded on NCBI^1^ with the registration number OK103600.1. The Ps3 gene sequence was compared with BLAST, and it was found that the Ps3 sequence was comparable with that of *Pseudomonas*, a genus with high similarity to Ps3 at 99.5%. In this study, appropriate gene sequences of strains were selected, and a phylogenetic tree was constructed (Supplementary Figure 4). The analysis showed that strains with higher homology to Ps3 were *Pseudomonas* sp. JC5 and *Pseudomonas protegens* CP-M2-5, which were located on the same branch. *Pseudomonas plecoglossicida* strain 2–3 was also found on a larger branch, with both of them in two separate branches.

### 3.2. Growth curve of algolytic bacteria

The growth curve of *Pseudomonas* sp. Ps3 was close to the “S” shape, and the model curve was shown in Supplementary Figure 5, which was well fitted with the actual growth condition of Ps3, with the correlation coefficient R^2^ = 0.992 (*p* < 0.001), indicating that the curve described the growth condition of *Pseudomonas* sp. Ps3 better. When combining the two, it was found that *Pseudomonas* Ps3 started to enter exponential growth after 2 h, the maximum rate of bacterial growth reached 1.01 h^−1^ at 5 h, and the bacterial density peaked at 10 h with a maximum number of 5.2 × 10^8^ CFU/ml then entered the death phase. The average growth rate of Ps3 reached 0.823 h^−1^.

### 3.3. Effects of different growth stages of bacteria on algal dissolution

**Figure 1:**
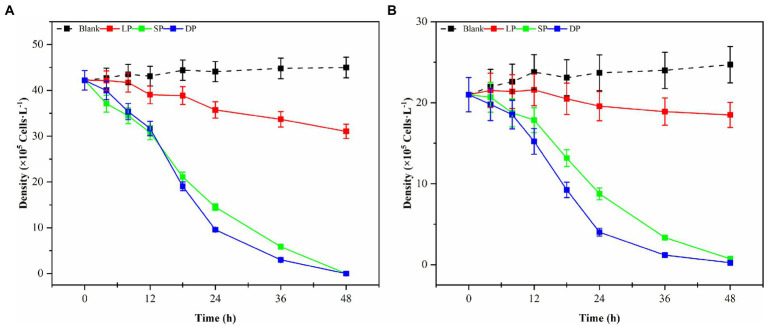
Changes in the density of *G. catenatum* **(A)** and *K. mikimotoi* **(B)** with time were observed during the different growth stages of Ps3 bacterial solution (LP-Logarithmic Phase; SP-Stationary Phase; DP-Death Phase).

**Figure 2:**
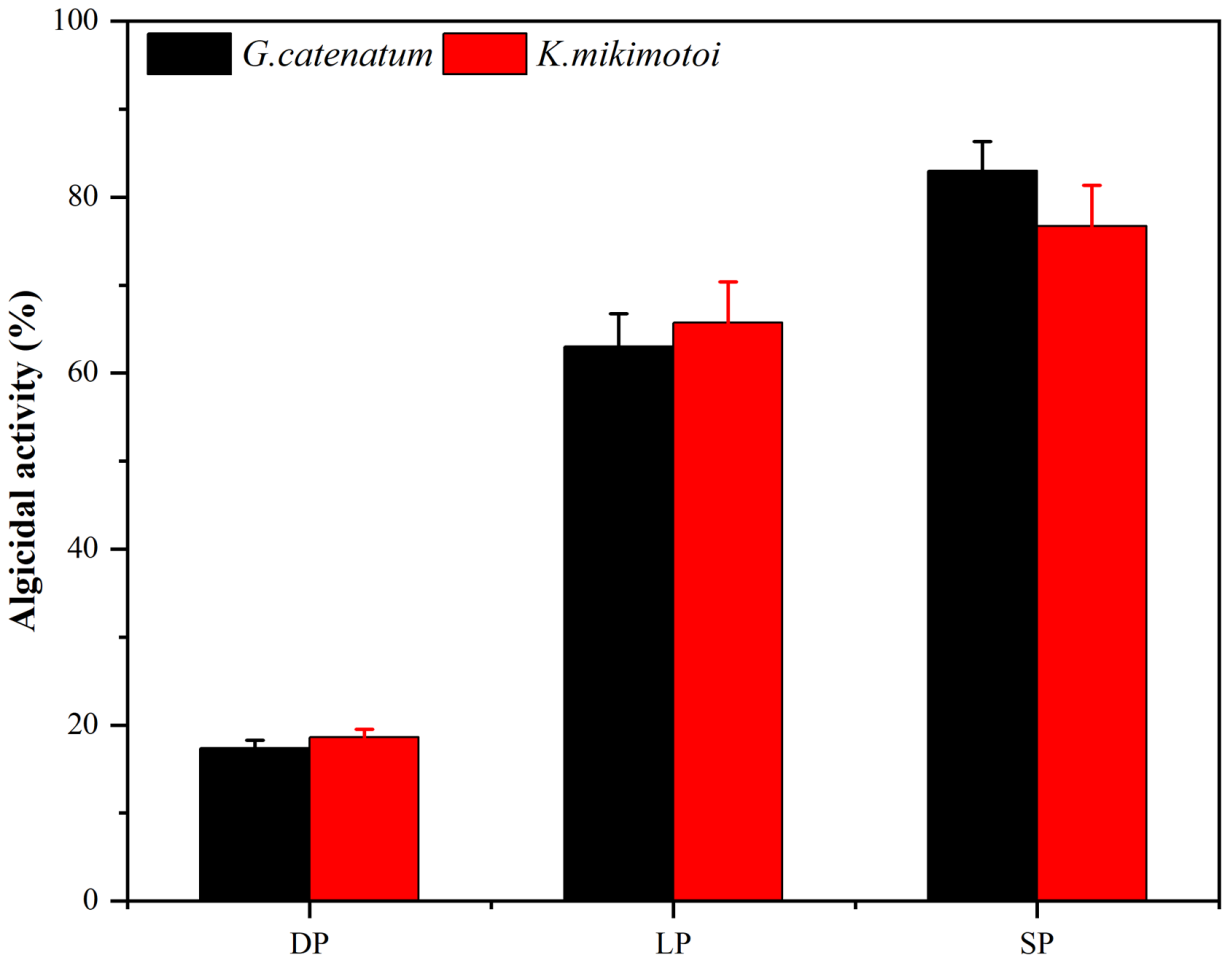
Comparison of the 24 h-algicidal activity of the growth bacterial solution at different growth phases (LP-Logarithmic Phase; SP-Stationary Phase; DP-Death Phase).

Bacterial numbers, growth activity, and metabolites vary during different growth stages, leading to notable differences in algal dissolution effects. Consequently, this study examined changes in algae density during the co-culture of Ps3 bacterial solution with two types of algae across various growth stages. The initial densities of *G. catenatum* and *K. mikimotoi* were 210 ± 15 cells/ml. After 48 h, the density of algae in the control group still increased. Compared with the control group, the density of *G. catenatum* and *K. mikimotoi* in logarithmic, stationary and death phases decreased by 17.0, 63.0, 83.0% at 24 h, and 25.0, 85.0, and 92.0% at 48 h (Figure 1). Observations indicate that while the bacterial solution cultured for 5 h can prevent the formation of *G. catenatum*, it is incapable of dissolving the algae. Prior to the 12-h mark, the bacterial solution exhibited a discernible inhibitory effect on the growth of *G. catenatum*, while after 12 h, the solution rapidly began to dissolve the algae. In a certain period of time, the bacteria in the stationary phase or death phase grow more fully, produce more active substances, and have a better effect on algae dissolution. It can be seen from Figure 2 that, by comparing the algae lysis effects of bacteria solution on the two kinds of algae in the death phase at different time points, the algae-dissolving effects of bacteria solution on the two types of algae reached 83.0 and 78.3% at 24 h, basically reaching the algae-dissolving effect. In 48 h, the algae could reach 85.1 and 92.0%, attaining the expected effect of alga dissolution. Based on the results obtained from the three experimental groups, it can be observed that the effect of fermentation broth in controlling *G. catenatum* was slightly more pronounced compared to that of *K. mikimotoi*.

### 3.4. Lytic effects of Ps3 on various red tide species

**Figure 3:**
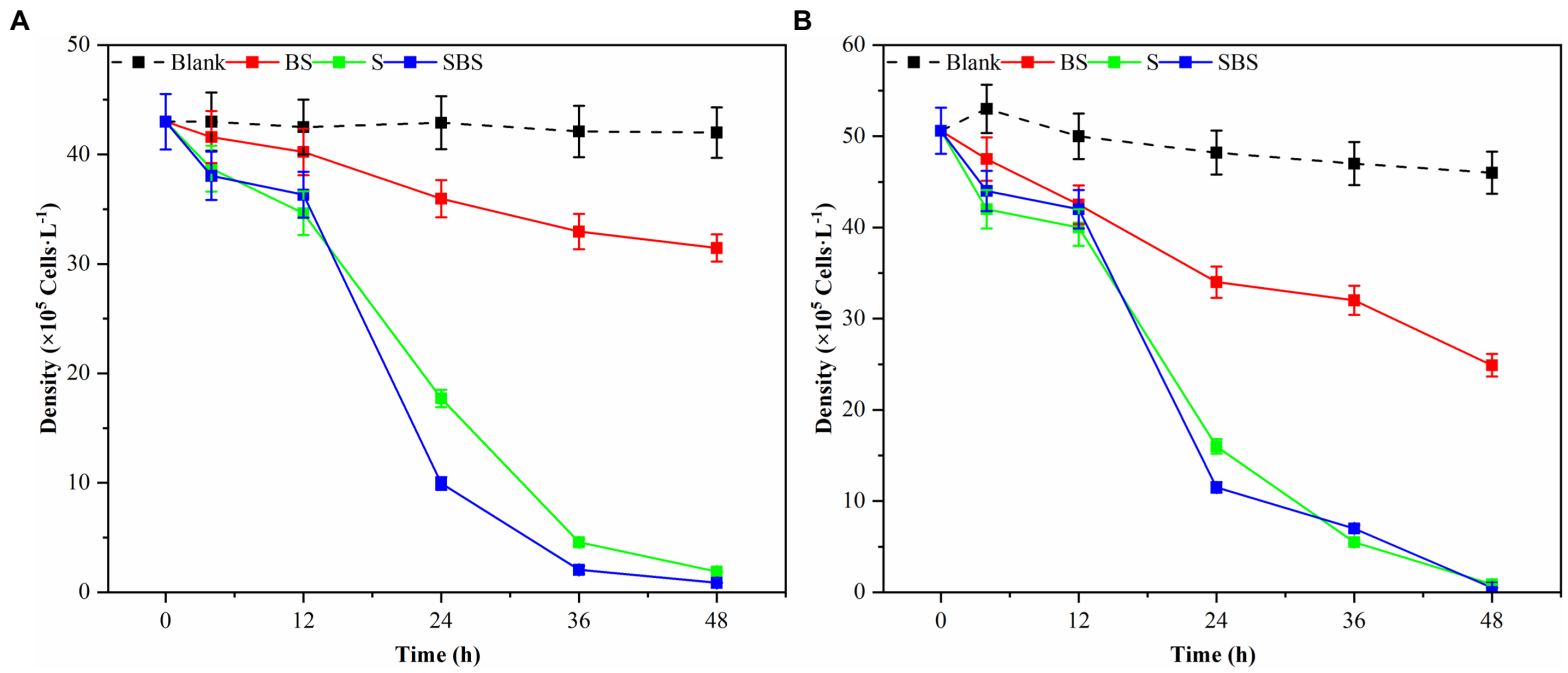
Trend of the number of different compositions of Ps3 on *G. catenatum* **(A)** and *K. mikimotoi* **(B)** [Ps3 broth (SBS); bacterial suspension (BS); *Pseudomonas* Ps3 (S)].

**Figure 4:**
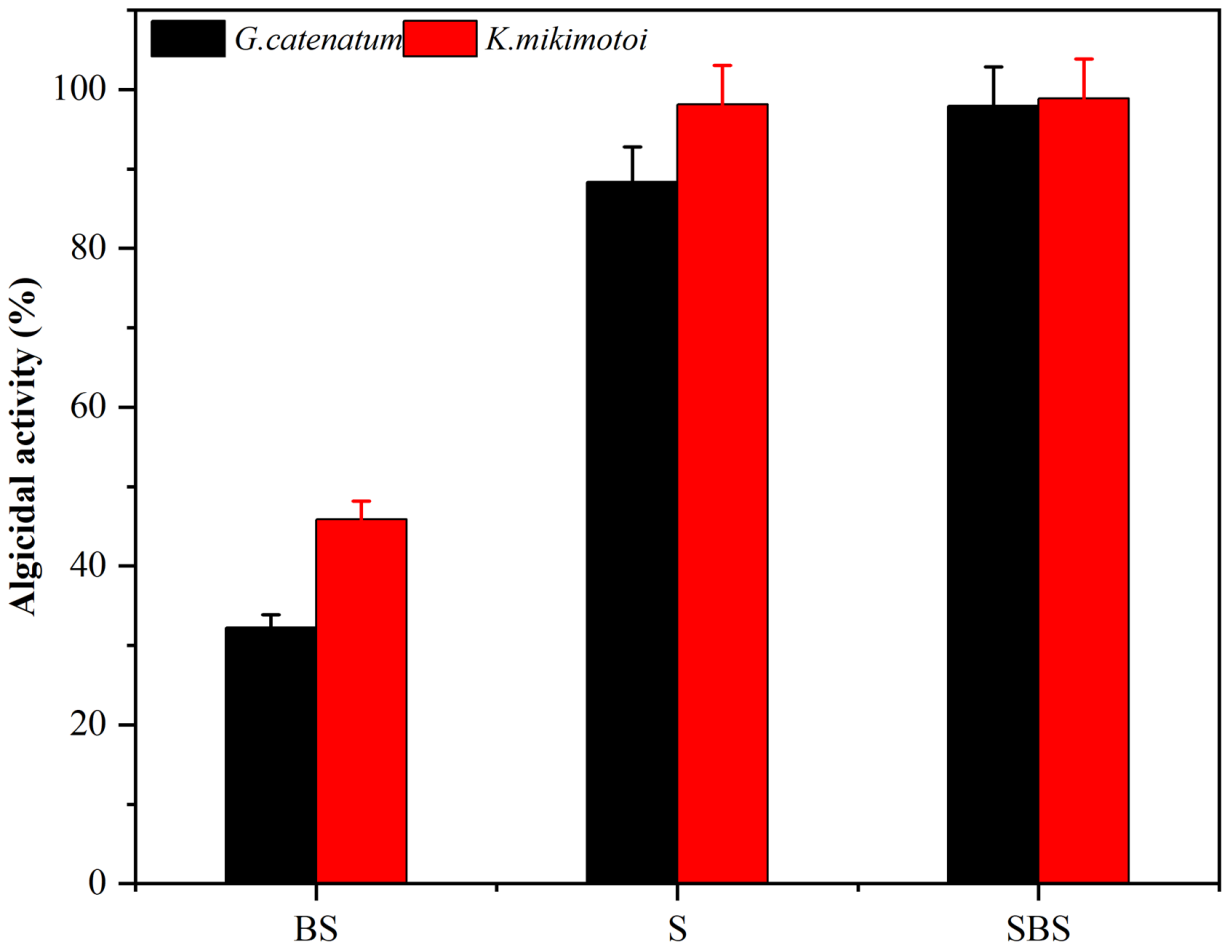
Algicidal activity of different composition to *G. catenatum* **(A)** and *K. mikimotoi* **(B)** of bacteria Ps3 at 48 h [Ps3 broth (SBS); bacterial suspension (BS); *Pseudomonas* Ps3 (S)].

Samples of supernatant, suspension, and primary bacteria were added to the algae solution at a rate of 2.0%, and their respective algolytic effects on both types of algae were monitored, as depicted in Figure 3. After 48 h in the control group, the density of *G. catenatum* and *K. mikimotoi* were 420 and 460 cells/ml, respectively, indicating a decrease of 4.5 and 14.5% compared to their initial densities. Notably, *G. catenatum* remained stable throughout the experiment, with its density increasing in the control group after 48 h. In contrast, the density of both algae species decreased significantly in the fermentation broth and bacteria liquid groups. Compared to the control group, the density of *G. catenatum* and *K. mikimotoi* decreased by 93.1 and 95.0%, and 90.4 and 93.5%, respectively, after 48 h. There was no significant difference between the two groups. In the 15°C group, the lysis rates at 48 h were 88.4, 97.9, 98.1, and 98.9%. It can be seen from Figure 4. That, after 48 h of inoculation and culture, the algal dissolution rate of bacterial liquid and sterile fermentation liquid reaches more than 95.0%, much higher than the algal dissolution rate of bacterial precipitation.

The observed effects strongly suggest that Ps3 fermentation liquid contains active components that inhibit algal cells, indicating that the algal dissolution mode of Ps3 is indirect. This is supported by the results shown in Figure 4, which demonstrate a significant inhibitory effect on algae by both the bacterial sediment suspension and the bacterial medium. This could be attributed to the growth of bacteria in the algal liquid and the secretion of active algal inhibitory components. As a result, the algal dissolution rates were 32.6 and 48.1% within 48 h.

### 3.5. Effects of different dosages of fermentation broth on red tide algae

**Figure 5:**
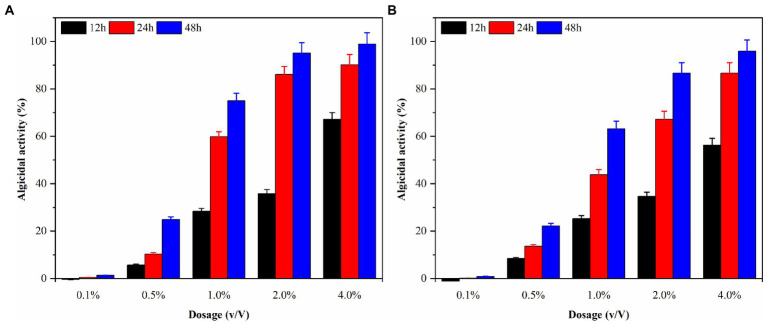
The algicidal activity of *G. catenatum* **(A)** and *K. mikimotoi* **(B)** with different dosage of sterile fermentation broth.

Local red tides tend to appear and dissipate rapidly, usually within 3–5 days. Therefore, this study aims to enhance the efficiency of algae dissolution and reduce the time required for it by modifying the experimental conditions. Consequently, sterile fermentation broth was supplemented with 0.1, 0.5, 1.0, 2.0, and 4.0% and inoculated into the algae solution of *G. catenatum* and *K. mikimotoi*. The bacterial medium served as the control group to evaluate the impact of the different amounts of supplemented fermentation broth on the effectiveness of algae dissolution, as illustrated in Figure 5. After 48 h, the density of *G.catenatum* and *K. mikimotoi* in the control group was 263 and 480 cells/ml, which decreased by 2.6 and 4.0%, respectively, compared to the initial experiment, and remained constant roughly throughout the investigation. When the fermentation broth was supplemented with 0.1%, there was no significant effect on the algae density. However, at 0.5%, it had a particular inhibitory impact. At 1.0%, the algae dissolution effect was direct. The 2.0 and 4.0% groups had a significant impact on dissolving both types of algae, with a more pronounced effect observed in *K. mikimotoi* than in *G.catenatum*. The 4.0% group significantly reduced the time required to dissolve the algae. The inhibition effect of Ps3 fermentation broth on *G.catenatum* and *K. mikimotoi* was positively correlated with the dosage, with a better inhibition effect observed at higher dosages.

### 3.6. Effect of ambient temperature on the effect of algae lysis in sterile fermentation broth

**Figure 6:**
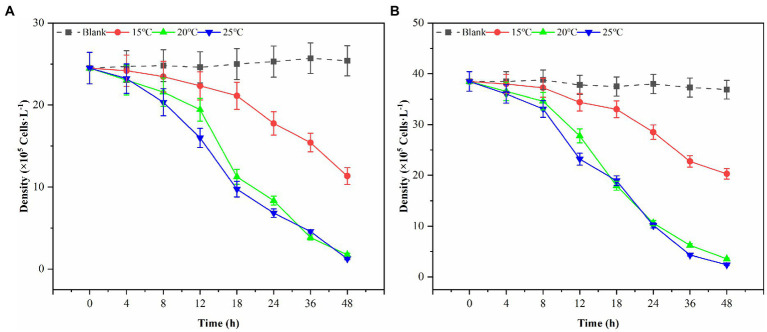
The changes of algal density of *G. catenatum* **(A)** and *K. mikimotoi* **(B)** with time were observed by the addition of Ps3 solution at different temperatures.

In this study, three possible temperature gradients (15°C, 20°C, and 25°C) were set up to observe Ps3 algae lysis during the red tide period in simulation. As shown in Figure 6, the algal density of the blank group generally remained unchanged at the end of the experiment for 48 h. Under the influence of 2.0% (v/v) of Ps3 fermentation solution, the density of *G. catenatum* decreased by 93.1 and 95.0% in the 20°C and 25°C groups, and the density of *K. mikimotoi* decreased by 90.4 and 93.5%. The fermentation solution had a good algae lysis effect on both kinds, and there was no significant difference between the 20°C and 25°C groups (*p* < 0.05). As for the 15°C group, the algae lysis rate was 55.3 and 45.0%, and Ps3 has a higher rate of algal lysis for *G. catenatum* than for *K. mikimotoi*.

### 3.7. Morphological changes of algal cells during algal lysis

**Figure 7:**
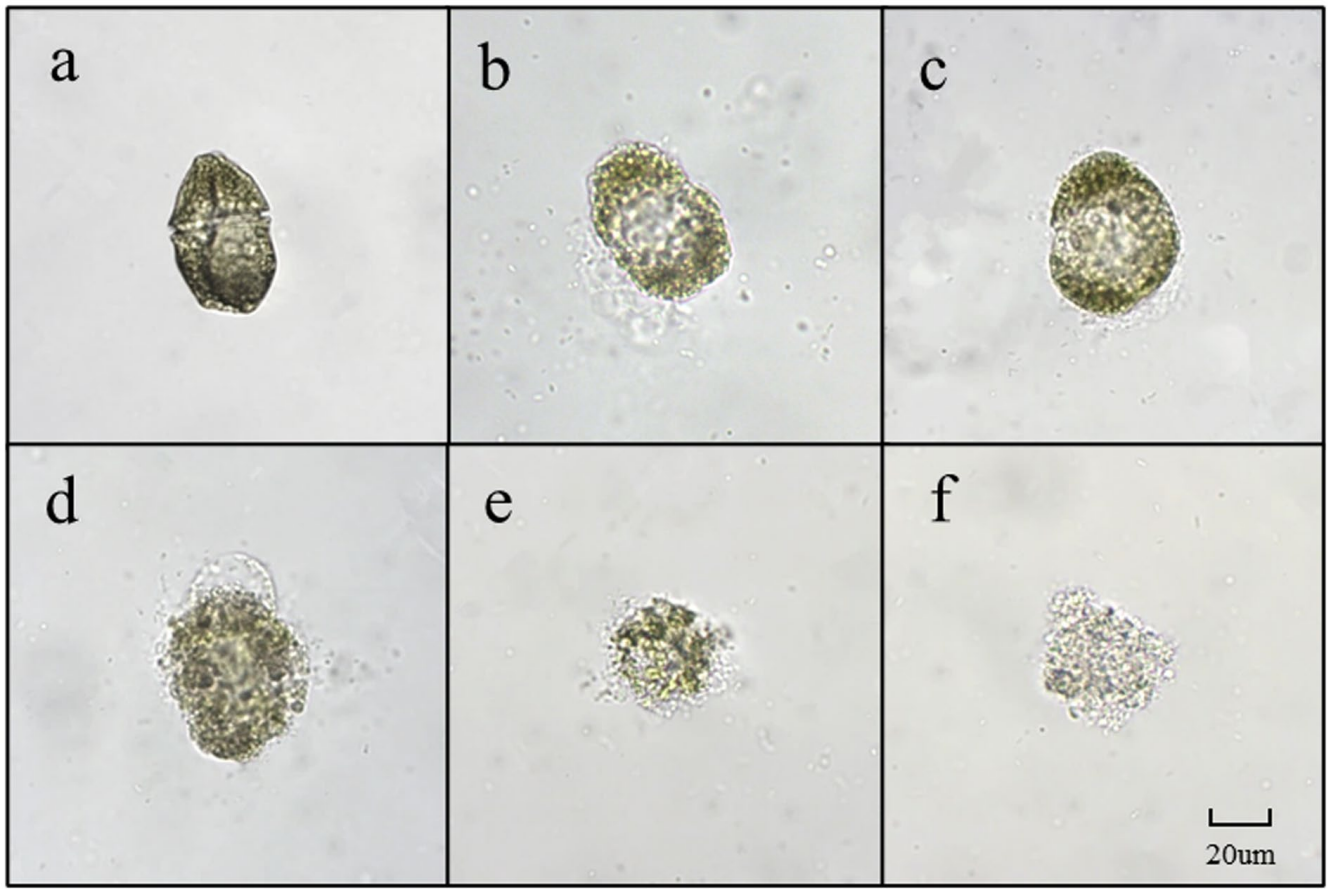
The main forms of algae in the algae dissolution process of *G. catenatum* under the influence of *Pseudomonas* sp. Ps3 sterile fermentation broth (**A–F** represent different stages of dissolution process, respectively).

Dinoflagellates are relatively small in size, with a diameter of 20–40 μm. As depicted in Figure 7A, *G. catenatum* exhibited good cell growth and distinct transverse grooves during the initial stages of bacterial-algal co-culture. Figure 7B illustrates that after 12 h of co-culture, the transverse sulcus of algae cells in the field of vision became unclear, granulation appeared inside, and irregularity emerged at the edge. Following 24 h treatment, the interior of the algae cells became hollow, a small amount of cytoplasm flowed out, and numerous cells died (Figure 7C). In Figure 7D, after 18 h, many algal cells still contained pigments, but their cell membranes were damaged, cytoplasm flowed out, and the cells were considered dead due to their irregularity. Figures 7E,F depict the progressive decomposition of cells and the disappearance of chromophores.

**Figure 8:**
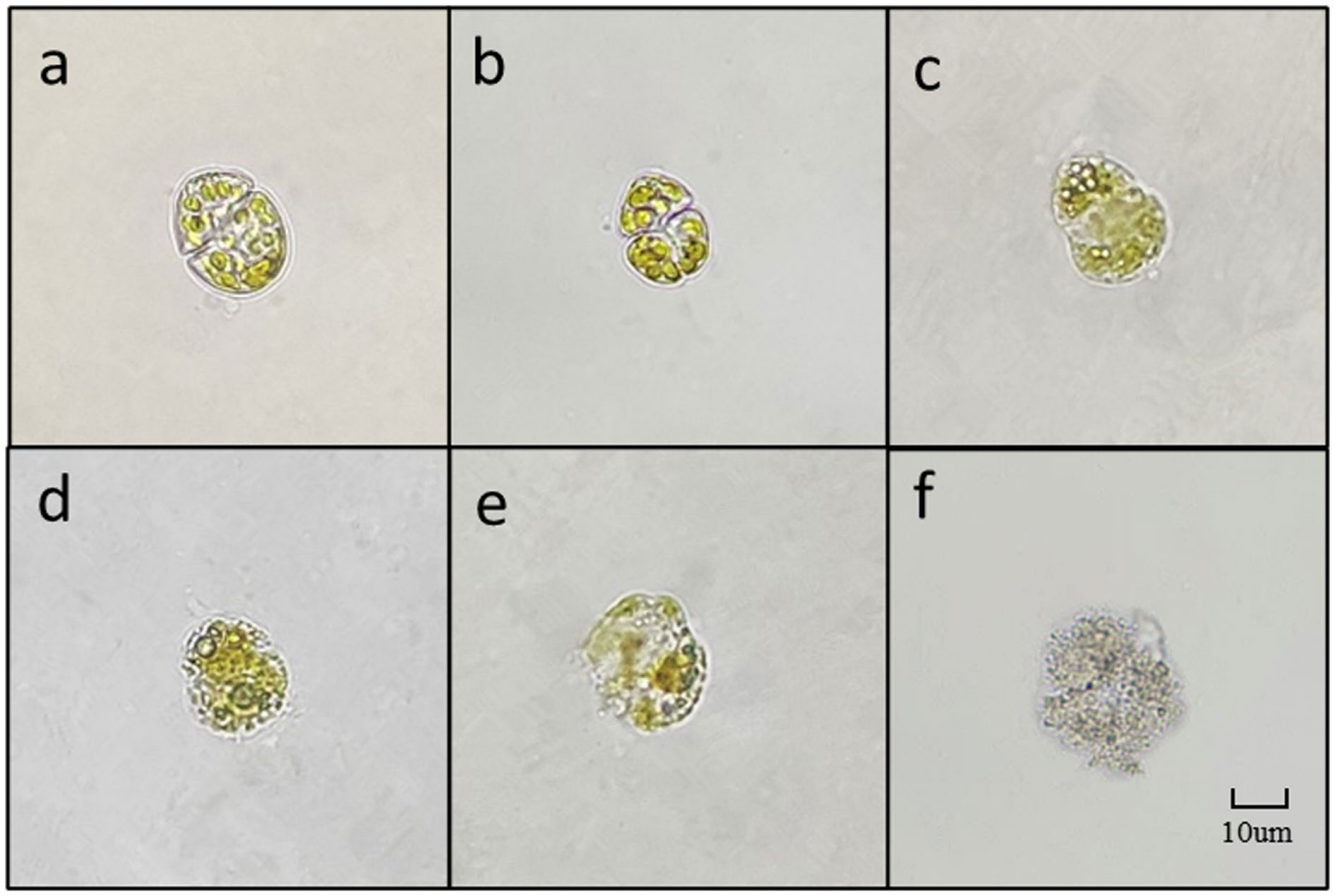
The main forms of algae in the algae dissolution process of *K. mikimotoi* under the influence of Ps3 sterile fermentation broth (**A–F** represent different stages of dissolution process, respectively).

As depicted in Figure 8, due to the small diameter of *K. mikimotoi* (3–6 μm), observing the algae cells at 400 times magnification is challenging. However, cell membrane deformation, blurred broken edges, and impaired integrity are apparent (Figure 8D). The cells then undergo deformation and become granulated (Figure 8E). The chromatophores become dark, and the cell contents diffuse, leading to the fading of chromatophores. Ultimately, the entire cell disintegrates into extremely fine particles, which become blurred and invisible (Figure 8F).

### 3.8. Algal dissolution of the crude separation liquid of bacterial fermentation liquid

**Figure 9:**
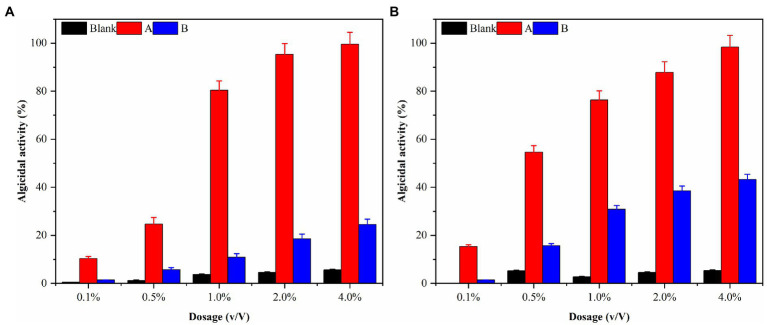
The algal lysis of *G. catenatum* **(A)** and *K. mikimotoi* **(B)** by ethyl acetate extraction phase **(A)** and residual phase **(B)** in fermentation broth of *Pseudomonas* sp. Ps3.

The densities of algal cells in *G. catenatum* and *K. mikimotoi* remained similar and did not exhibit any significant changes during a 48-h period after introducing varying volume ratios of ethyl acetate. As such, the impact of ethyl acetate in the algal lysis experiment can be considered negligible. The experimental results were shown in Figure 9A after different volume ratios of ethyl acetate extracted phase solution and residual phase solution was injected into the *G. catenatum*. The addition of a 4.0% (v/v) extraction phase solution resulted in a significant increase in the algae lysis rate, reaching 98.4%. Conversely, the use of a 4.0% (v/v) residual phase solution led to a low algae lysis rate of only 24.5% after 48 h, suggesting a poor capacity for algae lysis in the residual phase.

*K. mikimotoi* (Figure 9B) exhibited higher algae lysis rates of 15.4 and 54.7% when treated with 0.1 and 0.5% (v/v) ethyl acetate extraction phase solutions, respectively, compared to the *G. catenatum*, indicating a positive effect. The use of a 4.0% (v/v) ethyl acetate extraction phase solution significantly increased the algae lysis rate to 95.9%, exceeding the rates of other groups. Importantly, unlike the *G. catenatum* group, the residual phase solution with a volume fraction of 4.0% also significantly enhanced the algae lysis rate of Karenia mikimotoi to 45.0%, probably due to some differences in the algae species as well as the mechanism of algae lysis. *Pseudomonas* sp. Ps3 is mainly present in the ethyl acetate extraction phase, but the residual material still has a certain effect on algae lysis.

It can be seen that most of the active substances in the fermentation broth of Ps3 bacteria are lipid-soluble substances, which can be extracted by ethyl acetate. Gas chromatography–mass spectrometry (GC–MS) was used to analyze the structure of the algolytic active substances. Chromatograms of volatile components of ethyl acetate extract of Ps3 are shown in Supplementary Figure 6. The peak of the sample was accurately detected by GAS chromatography–mass spectrometry, and the baseline was stable. The volatile components of ethyl acetate extracted phase of Ps3 had more peaks, and the main retention time was between 16 and 26 min. The molecular ion peaks of the higher substances in the content of algal soluble active substances were 20.507, 18.256, 24.285, and 22.349 min. Within the ethyl acetate phase of Ps3 fermentation broth, there were seven absorption peaks observed between 16 and 20 min. Among these peaks, the cyclic (leucine-leucine) dipeptide (20.518 min) was the most prevalent, comprising 34.07% of the total. Several acids and esters exhibit cyclic structures, including the following: cyclic (dipeptides proline-leucine) (with a retention time of 18.266 min and a relative abundance of 25.47%), cyclic (L-leucanyl-L-phenylalanyl) (with a retention time of 24.285 min and a relative abundance of 10.47%), cyclic (phenylalanine proline) (with a retention time of 22.349 min and a relative abundance of 7.88%), and cyclic (glycylyl prolyl) (with a retention time of 16.425 min and a relative abundance of 4.69%). Additionally, 4-heptanone dihydrazone (with a retention time of 17.351 min and a relative abundance of 2.78%) and DL-alanyl-L-leucine (with a retention time of 16.12 min and a relative abundance of 2.18%) exhibit cyclic structures. The results indicate that the ethyl acetate extract of the fermentation broth of *Pseudomonas* Ps3 mainly contains cyclic dipeptides.

### 3.9. Biotoxicity of the bacterial fermentation broth

**Figure 10:**
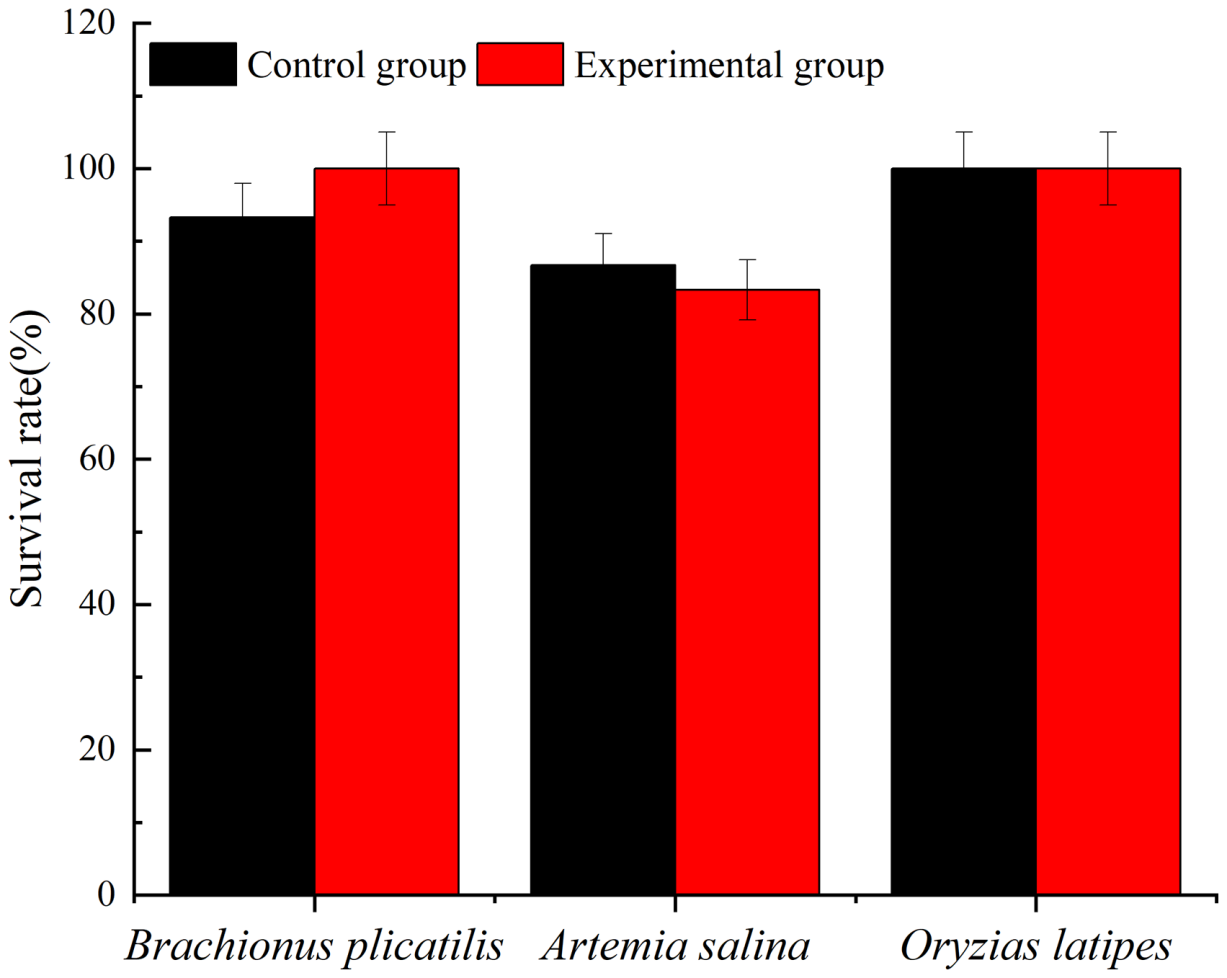
The proportion of living organisms.

The addition of 2.0% (v/v) Ps3 bacterial fermentation solution had little effect on the survival of all experimental organisms, but different organisms had varying sensitivities to toxicity. At 48 h, the survival rates of the control group’s *Brachionus plicatilis*, *Artemia salina*, and *Oryzias latipes* were 100, 83.3, and 100%, respectively. The survival rates of the experimental group’s *Brachionus plicatilis*, *Artemia salina* and *Oryzias latipes* were 93.3, 86.7, and 100%, respectively (Figure 10). There was no significant difference in the survival rates of each experimental organism compared to the control group.

## 4. Discussion

In the open ocean, there is a noticeable correlation between the behavior of bacteria and marine dinoflagellates. Specifically, there is a connection between the biomass of dinoflagellates and bacteria that can be observed (Fuhrman and Azam, 1980). Although there has been significant research on microalgae and microbe communities, there has been little focus on examining the interactions between these species at a species-specific level (Bratbak, 1985; Mayali and Azam, 2004; Rooney-Varga et al., 2005). Several studies have also shown that marine bacteria are capable of promoting or inhibiting microalgae growth (Iwata and Barber, 2004; Rooney-Varga et al., 2005).It has been shown that algal blooms associated with coastal ecosystems are mostly dominated by *Alphaproteobacteria*, *Gammaproteobacteria*, and *Bacteroidetes* (Teeling et al., 2012). The death of microalgae in coastal waters, caused by bacteria such as Ps3, can impact the growth rate of the phytoplankton population (Meyer et al., 2017). This phenomenon can potentially contribute to the sudden disappearance of algae blooms in the marine environment. The destruction of microalgae by heterotrophic bacteria is a possible mechanism that could regulate primary productivity in the surface ocean. *Pseudomonas* sp. is an effective biological control agent because it is generally believed to be harmless, non-pathogenic, and poses low risk to the environment, wildlife, and humans (Lee et al., 2011; Chen et al., 2015; Anderson and Kim, 2018).

Non-specific inhibition of diatom growth by *Pseudomonas* sp. has also been observed with *Peridinium bipes*, and *Anabaena cylindrica* (Kang et al., 2007; Jung et al., 2012). A report show supernatants of cultures of *Pseudomonas putida* can kill algae without coming into direct contact with the algae by secreting algicidal substances (Zhang H. et al., 2011). Harmane was isolated in *Pseudomonas* sp. as a kind of algicidal substance. Cultures of K44-1 at a low concentration inhibited *Anabaena* sp. and *Oscillatoria* sp. effectively (Kodani et al., 2002). Additionally, *Pseudomonas* sp. provided a particularly effective inhibitory effect on diatoms. According to the study by Zhang H. et al. (2011), *Stephenodiscus hantzschii* has been identified as a common diatom in winter algae blooms. When 5.0 × 10^6^ cells/ml were used, the inhibition rate of *Pseudomonas fluorescens* reached 90.0%, explaining the disappearance of diatoms (Kim et al., 2007; Jung et al., 2008). Furthermore, the cellulose fermentation *Pseudomonas fluorescens* displayed better algal control ability when compared with suspended free cells (Kang et al., 2012). The algicidal compounds rupture microalgae cells and lead to the release of more labile organic matter.

In this study, we demonstrate for the first time that Ps3 has high algicidal activity, and therefore, it has high potential as a bio-agent against HABs. The results indicate that Ps3 fermentation solution’s soluble algae has a highly significant effect when added at 2.0 and 4.0% quantities, whereas the addition of 0.1 and 0.5% of soluble algae resulted in poor performance. One possible explanation for the low rate of algae lysis is that the inclusion of algal-inhibiting active components is less effective. These findings provide further evidence supporting the notion that Ps3 inhibits algae growth by secreting active substances. In the process of algal dissolution, the change of algal morphology reflects a possible mechanism for algal dissolution. Based on research, the algicidal properties of Ps3 fermentation broth on *G.catenatum* appear to involve damaging the transverse furrow, which subsequently leads to cytoplasm liquefaction and the loss of fundamental cellular functions. Ultimately, the emulsification of the cell membrane and wall occurs, while the chloroplasts undergo decomposition, resulting in discoloration. Studies have shown that the *K. mikimotoi* dissolution process mainly affects the loss of cell membrane integrity, rapid cell rupture, inducing reactive oxygen species (ROS), lipid peroxidation, and photosystem II (PSII) inhibition, and ultimately destroys the subcellular structure, leading to the death of algal (Li et al., 2017). The primary factor that may account for the contrasting effects of Ps3 fermentation broth on *G.catenatum* and *K. mikimotoi* is likely to be their distinct cell size and response mechanisms. In future research, further exploration will be conducted on the relevant mechanisms.

Meanwhile, in the investigation of the impact of environmental temperature on the sterile fermentation solution’s ability to lyse algae, it was observed that the Ps3 fermentation solution’s efficiency in dissolving algae significantly decreased under lower experimental temperatures. This reduction in efficiency could be attributed to the lowered activity of algae at low temperatures and decreased exchange of substances between the algae and the external environment, which ultimately led to a reduction in the lytic effect of the fermentation solution (Li et al., 2021).

A toxicity testing experiment was conducted on waterborne organisms using Ps3 fermentation solution to evaluate its effectiveness as an algae control method. The results revealed that this approach has minimal toxicity to aquatic organisms as no unusual growth was observed during the experiment. This indicates that it is a relatively safe method for controlling algae. However, further research is necessary to examine any potential toxic effects. The use of algicidal bacteria to control red tides may result in elevated nutrient levels in the surrounding water. This is due to the release of nutrients from various sources, including the deceased red tide algal cells (Tilney et al., 2014). As a result, any remaining nutrients may promote the regrowth of harmful algal blooms. As such, when utilizing *in situ* methods for controlling red tides, it is important to carefully monitor and manage changes in nutrient levels within the ocean environment. Overall, this method has promising applications for controlling red tides in aquaculture water bodies. The findings suggest that utilizing Ps3 as a bio-controller for reducing or eliminating harmful algae blooms is a cost-effective and sustainable approach with long-term benefits. This *in situ* strategy has greater potential to suppress harmful algal blooms compared to existing methods.

The algal control system successfully eliminated harmful algae from the marine environment using a combination of bacteria, primarily *Pseudomonas* sp. and *Bacillus* sp., which were found to be the most effective in the process of algicidal activity. To achieve optimal results, it was necessary to optimize the fermentation conditions for these bacteria, in order to increase the production of algicidal compounds to the highest level possible (Kim et al., 2015; Hu et al., 2019). Algicidal bacteria face challenges surviving in the complex and unstable marine environment, and may also be inefficient at producing algicidal compounds (Hu et al., 2019; WANG et al., 2021). To mitigate algae blooms using algicidal bacteria, the algicidal compounds were extracted from a fermentation broth that had been optimized for this purpose. Previous studies have reported that several amino acids possess the capability to inhibit algae growth. For instance, a study found that *Streptomyces phaeofaciens* S-9 secretes L-lysine, which can disrupt cyanobacterial cells (Yamamoto and Harayama, 1998). Moreover, the amino acid L-lysine has been observed to exhibit algicidal activity against *Microsystis* cells (Hehmann et al., 2002). We ruled out the possibility of natural amino acids serving as algicidal metabolites from Ps3. This was primarily because Ps3, which was used as a control, exhibited significant algicidal activity despite having the highest concentration of amino acids in the supernatant of its culture. Our experiments suggest that the algicidal activity of Ps3 can be attributed to the presence of natural amino acids within the substance. As a result, it is necessary to perform certain pre-treatments, such as immobilization, to effectively gather the compounds and create a controlled-release formula (Kang et al., 2012; Ni et al., 2015), prior to the dispersion of algicidal compounds. It is important to acknowledge that implementing this HAB-control strategy would also result in an increase in the complexity and cost of biological products. In the fermentation liquid of Ps3, an observable rise in intracellular content was detected within the first 24 h, followed by a stabilization of intercellular content (unpublished data). There seemed to be a dose–response correlation between the fermented liquid and intracellular contents. The rapid escalation in intracellular content is linked to the degree of membrane lipid peroxidation and the intensity of cell membrane lysis (Wang et al., 2011). These findings suggest an immediate strengthening of microalgae antioxidant enzyme activity in response to *P. aeruginosa* damage. Numerous factors, such as toxicant accumulation, can cause a decline in antioxidant enzyme activity, as this toxic substance can harm the cytomembrane system and pigment synthesis of cells (Qian et al., 2008; Zhang F. et al., 2011). To summarize, this report establishes the significant algicidal activity of strain Ps3, which is considered a non-pathogenic bacterium and environmentally safe. Thus, Ps3 has the potential to be an effective bio-controller for harmful algal blooms. Moreover, it was observed that strain Ps3 exhibited greater algicidal activity in all growth stages when grown under fermentation conditions compared to co-culture conditions. This discovery suggests that it may be feasible to manipulate oxygen levels during bacterial growth to improve algicidal activity and achieve greater control over harmful algal blooms. In upcoming studies, the use of clay may also be investigated as a potential complement to algicidal bacteria for red tide control.

## 5. Conclusion

The red tide dinoflagellate exhibited significant impairment when exposed to Ps3 at a fermentation liquid dosage exceeding 5.0% (v/v). High doses of co-culture and fermentation liquid induced abnormal cellular metabolism in the dinoflagellate, including the suppression of its defense system against ROS. Although the intracellular enzyme levels increased in response to the Ps3 fermentation liquid, the red tide cell membrane still underwent decomposition, and there was an excessive accumulation of intracellular contents. Based on these observations, as well as the morphology and structural analysis of the red tide cells, it is suggested that Ps3 primarily exerts its algicidal effects through oxidative damage and membrane destruction. Further investigation is needed to ascertain whether the accumulation of ROS or the impaired antioxidant system is accountable for the algal lysis. This study explored the mechanisms and potential bioactive compounds of *Pseudomonas* Ps3 for algae lysis. In future endeavors, *Pseudomonas* Ps3 could serve as the central element for optimizing the implementation conditions in the field, leading to enhanced efficiency in algal lysis.

## Data availability statement

The datasets presented in this study can be found in online repositories. The names of the repository/repositories and accession number(s) can be found at: https://www.ncbi.nlm.nih.gov/genbank/, OK103600.1.

## Author contributions

LZ wrote the manuscript. HL, YW, and GY performed the data collection and the data collection. YS conceived the idea, supervised the work, and provided bacterial strains. All authors contributed to the article and approved the submitted version.

## Funding

This work was supported by the National Key Research & Development Plan “Strategic International Scientific and Technological Innovation Cooperation” (2016YFE0202100), Marine Red Tide Early Warning and Prevention in Pingtan coastal area (PT2021006), National Natural Science Foundation of China (41573075), and Fujian Provincial Water Conservancy Technology Project (SC-292, DH-1558, 21NB000922, and MSK202202).

## Conflict of interest

The authors declare that the research was conducted in the absence of any commercial or financial relationships that could be construed as a potential conflict of interest.

## Publisher’s note

All claims expressed in this article are solely those of the authors and do not necessarily represent those of their affiliated organizations, or those of the publisher, the editors and the reviewers. Any product that may be evaluated in this article, or claim that may be made by its manufacturer, is not guaranteed or endorsed by the publisher.

## Footnotes

^1^ http://www.ncbi.nlm.nih.gov
