# Supplementary material for: A novel algicidal properties of fermentation products from Pseudomonas sp. Ps3 strain on the toxic red tide dinoflagellate species
Source: Front Microbiol. 2023 Apr 17;14:1146325. doi: 10.3389/fmicb.2023.1146325 (PMC10150927; doi:10.3389/fmicb.2023.1146325)
Supplement: Supplementary file 1 [file Data_Sheet_1.docx]

**A Novel Algicidal Properties of Fermentation Products from *Pseudomonas* Sp. Ps3 Strain on the Toxic Red Tide Dinoflagellate Species**

Luwei Zheng ^1^, Hong Lin^1^, Barathan Balaji-Prasath ^1,2,3^, Yu Ping Su ^1 2,3,*^, Ying Wang^1^, Yi Zheng ^4^, Guanglang Yu ^1^

^1^College of Environmental and Resource Science, College of Carbon Neutral Modern Industry, Fujian Normal University, Fuzhou 350007, PR China

^2^Fujian Key Laboratory of Pollution Control and Resource Recycling, Fuzhou 350007, PR China

^3^Fujian Province Research Centre for River and Lake Health Assessment, Fuzhou 350007, PR China

^4^Fujian Key Laboratory of Special Marine Bio-resources Sustainable Utilization, Fujian Normal University, Fuzhou 350007, P. R. China

Correspondence: ypsu@fjnu.edu.cn (Y.P.S.)


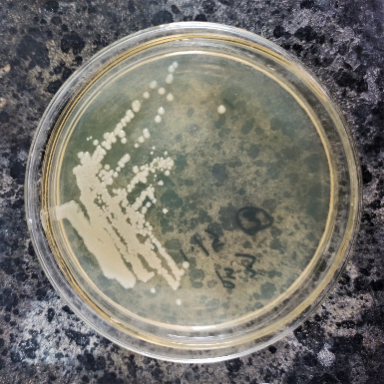


Supplementary Fig.1 The basic morphology of *Pseudomonas* Ps3


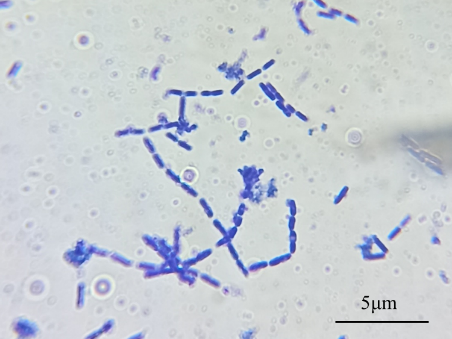


Supplementary Fig.2 Gram staining of *Pseudomonas* Ps3


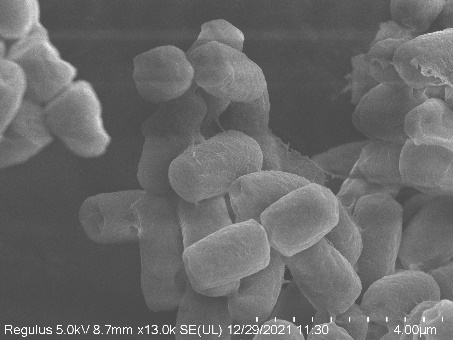


Supplementary Fig.3 Scanning electron microscopy (SEM) results of *Pseudomonas* Ps3


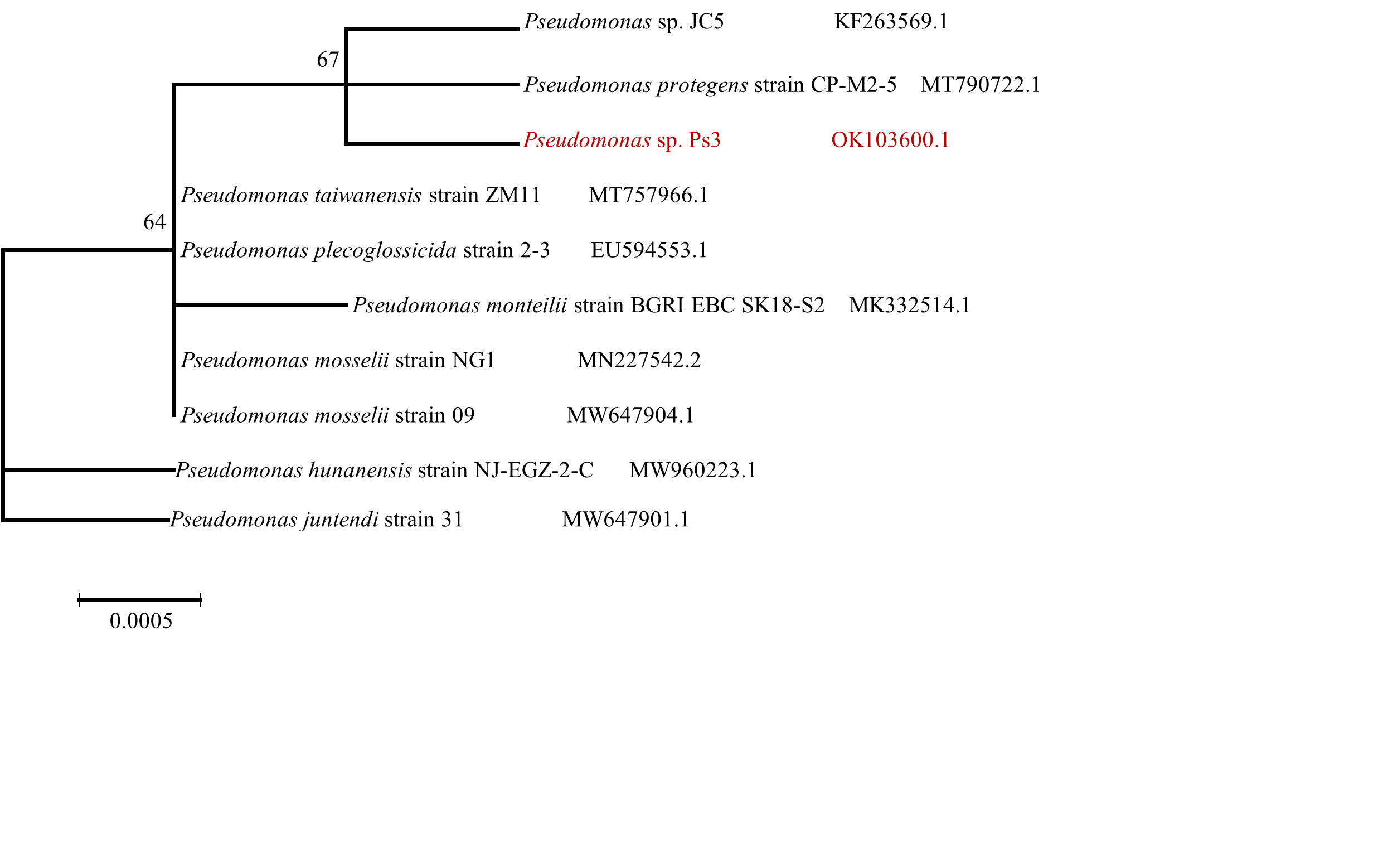


Supplementary Fig.4 Phylogenetic trees of *Pseudomonas* Ps3 based on 16S rDNA gene sequence analysis

**
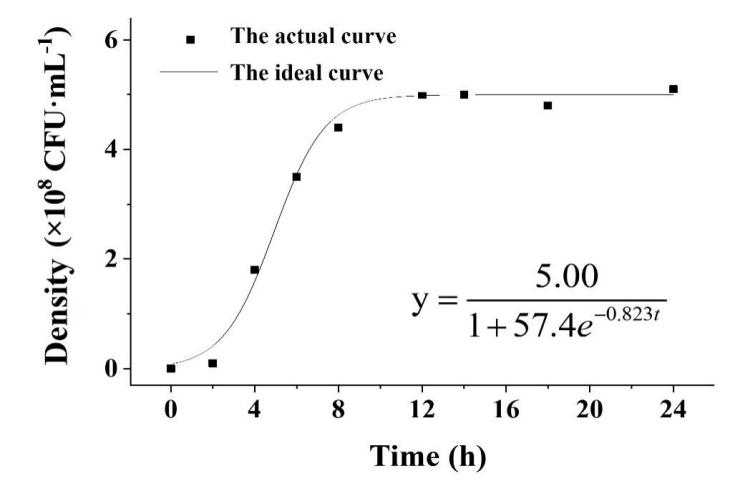
**

Supplementary Fig.5. The fitting curve of *Pseudomonas* sp. Ps3 growth status and growth dynamics


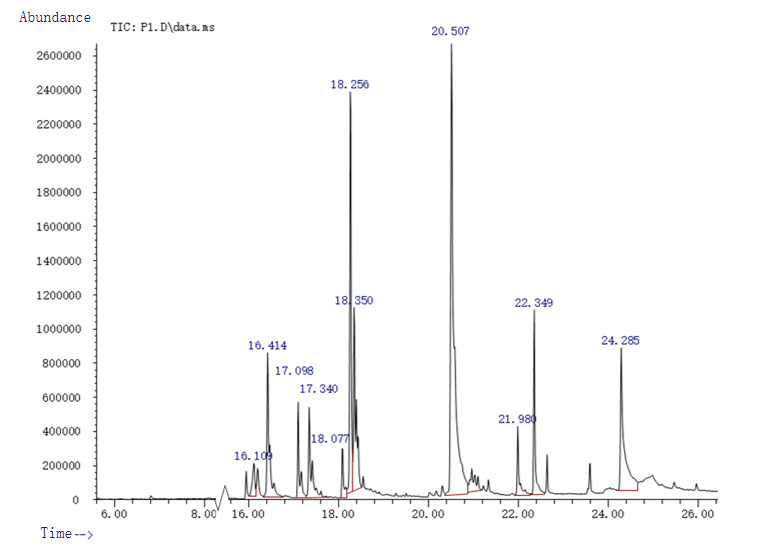


Supplementary Fig.6. Characterization of the algicidal compound secreted by fermentation broth and its molecular weight was validated using GC-MS.
